# Case Report: Differential diagnosis of hematuria in the emergency department: emphasizing double J stent-inferior vena cava fistula

**DOI:** 10.3389/fmed.2025.1570823

**Published:** 2025-04-30

**Authors:** Wenqi Qi, Shou-Yin Jiang

**Affiliations:** ^1^Department of Emergency Medicine, Second Affiliated Hospital, Zhejiang University School of Medicine, Hangzhou, China; ^2^Zhejiang Key Laboratory of Trauma, Burn, and Medical Rescue, Hangzhou, China; ^3^Zhejiang Province Clinical Research Center for Emergency and Critical Care Medicine, Hangzhou, China; ^4^Research Institute of Emergency Medicine, Zhejiang University, Hangzhou, China; ^5^National Emergency Medical Rescue Base, Hangzhou, China

**Keywords:** hematuria, double-J stent, intravenous migration, case report, complication

## Abstract

**Introduction:**

Hematuria, a common clinical indicator of genitourinary tract pathology, arises from diverse etiologies including calculi, infections, malignancies, trauma, and iatrogenic causes. Initial evaluation requires hemodynamic assessment, identification of underlying causes, and urinary drainage optimization. This report highlights a rare case of iatrogenic hematuria secondary to double-J stent migration into the inferior vena cava.

**Case presentation:**

A Chinese male presented with acute left flank pain and gross hematuria persisting for 4 h. Diagnostic imaging revealed a left ureteral stone, prompting double-J stent placement at a local hospital. Despite intervention, hematuria worsened, necessitating abdominal CT. Imaging identified proximal migration of the left double-J stent into the inferior vena cava, with no evidence of vascular injury. Due to concerns regarding inadequate drainage and infection risk, conservative management without catheter clamping was initiated prior to referral. Definitive treatment involved ureteroscopic stent removal under direct visualization at our institution, resulting in rapid symptom resolution.

**Conclusion:**

This case emphasizes three critical clinical insights: (1) Persistent postoperative hematuria warrants consideration of iatrogenic causes, particularly following urologic device placement. (2) Imaging modalities, especially CT, are indispensable for detecting atypical stent migration. (3) Comprehensive history-taking must include prior urologic interventions to guide differential diagnosis. While double-J stent migration into major vessels remains exceptionally rare, its recognition prevents delayed management of potentially life-threatening complications. Clinicians should maintain heightened vigilance for device-related hematuria in patients with refractory symptoms post-procedurally, ensuring prompt imaging evaluation and multidisciplinary intervention when indicated.

## Introduction

Hematuria represents a common clinical manifestation that may originate from any anatomical site within the urinary system, including the kidneys, ureters, bladder, prostate, and urethra. Major etiological factors encompass urolithiasis, urinary tract infections, neoplasms, as well as iatrogenic and traumatic causes. Mechanical injuries ranging from blunt or penetrating trauma to iatrogenic damage are capable of inducing hemorrhagic manifestations throughout the urinary tract. Among these etiologies, ureteral injury constitutes merely 1% of all urinary system trauma cases (1). Notably, iatrogenic ureteral injuries predominantly occur during gynecologic, urologic, and colorectal surgical procedures, accounting for approximately 80% of all ureteral injury cases (2).

First introduced in 1978 by Finney, the double-J ureteral stent has emerged as a cornerstone intervention in urological practice and one of the most frequently deployed devices worldwide (3). While its expanding clinical application has revolutionized urinary tract management, this widespread adoption has been accompanied by reports of procedure-related complications. Most adverse effects, including infection, encrustation, and stent-associated discomfort, typically resolve spontaneously with conservative management. Of particular clinical significance are rare but life-threatening vascular complications such as endovascular stent migration. The inaugural documentation of venous ectopia was reported by Michalopoulos in 2002 following pyelolithotomy, yet such occurrences remain exceptionally uncommon in clinical literature (4).

This article details a clinical case of hematuria secondary to aberrant stent migration. Comprehensive diagnostic evaluation-encompassing meticulous history-taking, systematic physical examination, and advanced cross-sectional imaging-revealed the presence of a double-J stent within the inferior vena cava. Through rigorous differential diagnosis, the definitive etiology was identified as retrograde migration of the ureteral stent from the renal pelvis into the venous system via the renal vein. By synthesizing current evidence through a systematic literature review, this study seeks to enhance clinical recognition of rare vascular complications associated with double-J stents, and provide evidence-based insights to guide therapeutic decision-making.

## Case presentation

A 37-year-old male presented to our emergency department with acute-onset left flank pain and gross hematuria persisting for 4 h. The patient’s medical history revealed ureteral stent implantation for urolithiasis management 24 h prior at a peripheral institution. During the index event, he manifested progressive left flank and costo-vertebral angle tenderness accompanied by visible hematuria, with subsequent failure of symptom resolution during observational management at the initial facility.

It was informed that the abdominal computed tomography (CT) demonstrated aberrant positioning of the left ureteral stent, with proximal coil termination within the inferior vena cava lumen. Given concerns regarding compromised hematuria clearance and ascending infection risks associated with urinary diversion, the primary care team adopted a conservative approach-maintaining continuous urinary drainage via the indwelling catheter while arranging emergent transfer to our tertiary care center for endovascular intervention.

**Figure 1:**
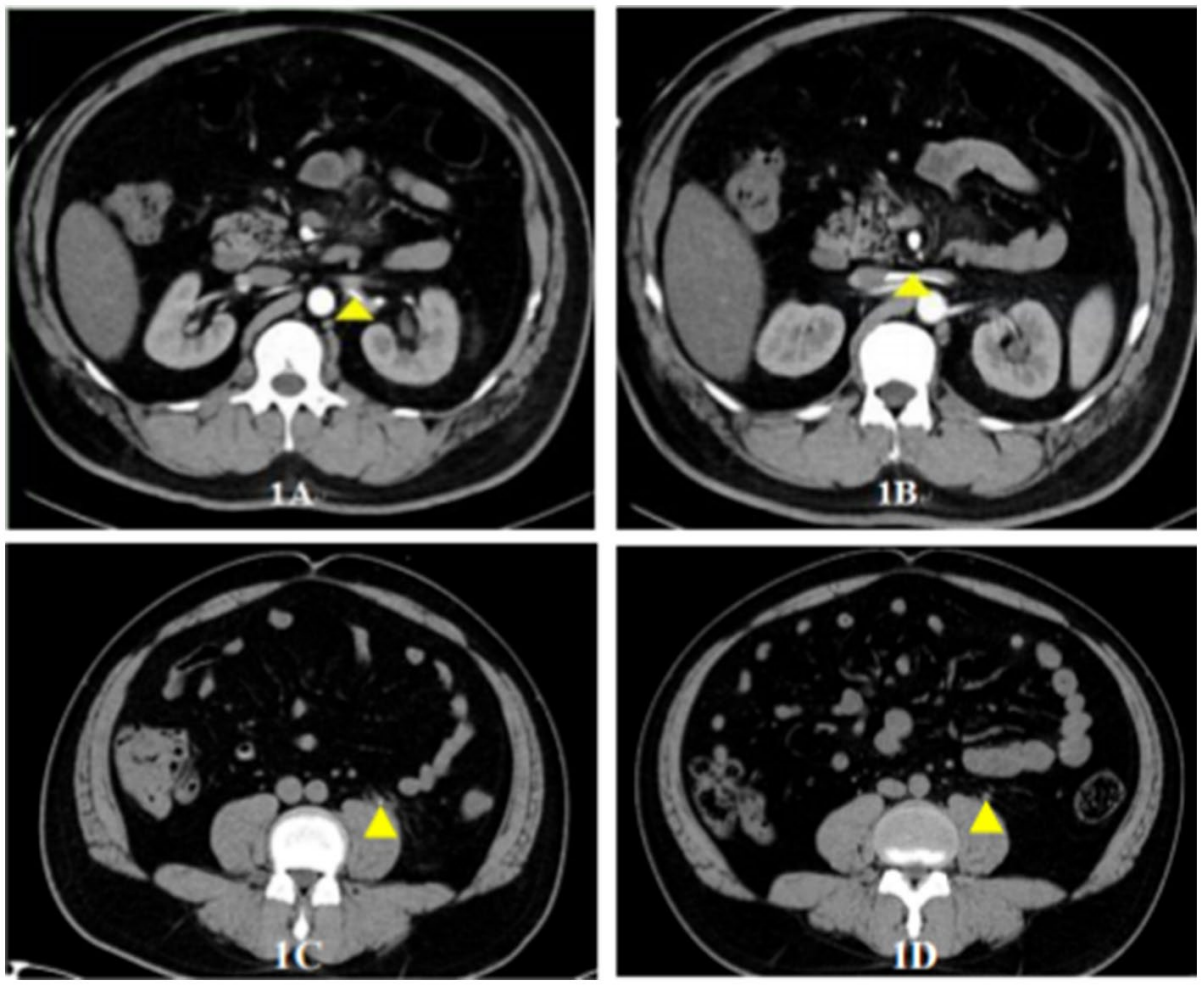
Radiographic documentation of double-J stent migration and associated urological changes. **(A)** Coronal CT reconstruction: Annotated marker (yellow triangle) demonstrates the aberrant trajectory of the double-J stent traversing from the left ureter into the left renal vein (Supplementary Video P1). **(B)** 3D vascular reconstruction: Sequential migration pathway visualized, showing stent progression from the left renal vein to the inferior vena cava (yellow triangular markers). **(C)** Axial CT at stent removal: Concurrent left ureteral calculus identified at the ureteropelvic junction (yellow annotation). **(D)** Follow-up CT at 2-week post-removal: Complete resolution of previously documented ureteral calculus, confirming stone elimination (yellow marker indicates original stone location).

Upon emergency department admission, the patient exhibited stable vital signs (temperature 36.8°C, BP 121/69 mmHg, HR 81 bpm) with intact neurological status. Physical examination revealed left quadrant abdominal tenderness without rebound, accompanied by characteristic left renal angle tenderness on percussion. Obvious bloody urine is visible within the urinary catheter, with the urine output manifesting as a pale red color. Laboratory analysis demonstrated leukocytosis (WBC 11.3 × 10^9^/L) with elevated inflammatory markers (IL-6 29.27 pg./mL, CRP 29.4 mg/L), while procalcitonin (0.15 ng/mL) and coagulation profiles remained normal. Urinalysis confirmed significant hematuria (1,381 RBCs/μL) with mild leukocyturia (11 WBCs/μL). Abdominal CT imaging delineated two critical findings: appropriate positioning of the left double-J stent and migration of its proximal segment through the left renal vein into the inferior vena cava (Figures 1A,B; Supplementary Video P1). Clinical evaluation systematically excluded alternative diagnoses including urinary tract infection (absence of fever/dysuria), urolithiasis (existing stent placement), and malignancy (negative imaging findings). The temporal correlation between recent stent placement (24 h prior) and symptom onset, combined with radiologically confirmed device migration, established the definitive diagnosis of iatrogenic hematuria secondary to double-J stent migration into the inferior vena cava.

Upon completion of the preoperative assessment, a multidisciplinary team, composed of specialists from urology, vascular surgery, and interventional radiology, established the indication for ureteroscopy. Taking into account the small caliber of the double-J stent, the minimal risk of vessel injury due to perforation, the low probability of substantial bleeding, and the fact that the distal end of the stent remained within the left ureter, a plan was formulated to extract the double-J stent under direct ureteroscopic guidance. The emergency procedure was successful in retrieving the left ureteral stent. Direct visualization through ureteroscopy revealed clear bilateral ureteral orifices, and the left ureter was clearly identified. The double-J stent was located and then removed. Post-removal, no bladder bleeding was detected, and the ureteroscope was withdrawn. Given the displacement of the ureter toward the inferior vena cava at that moment, re-placement could potentially elevate the risk of infection and further displacement. Thus, it was recommended that the patient undergo a follow-up abdominal CT scan 2 weeks later to determine the subsequent treatment plan. At that time, the patient’s ureteral stones had not induced obstructive hydronephrosis; hence, nephrostomy was not contemplated.

**Figure 2:**
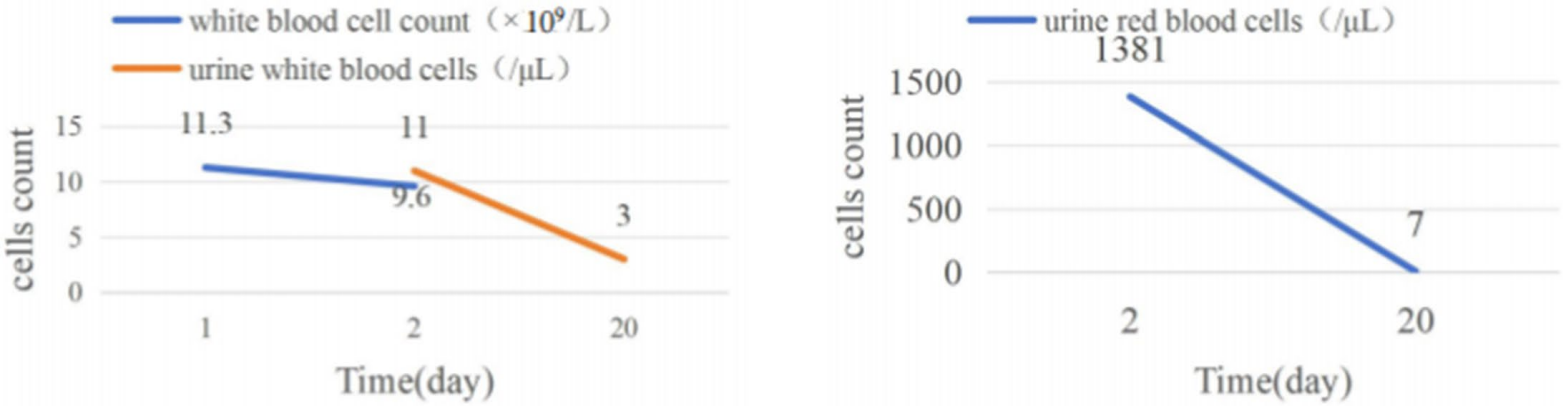
Dynamics of blood leukocyte counts, urinary leukocyte counts, and urinary erythrocyte counts preoperatively, postoperatively, and 2 weeks after double-J stent removal.

Following the operation, the patient’s hematuria resolved, and there was no occurrence of fever. There was no percussion pain in the bilateral ureteral tracts and renal areas. A follow-up abdominal CT scan indicated the presence of a left upper ureteral stone, accompanied by mild dilation and hydronephrosis, as well as exudate in the vicinity of the left urinary tract (Figure 1C). The hemoglobin level remained steady at 144 g/L, and the white blood cell count was 9.6 × 10^9^/L (Figure 2). Urinalysis results showed that the urine was light red, with 111 red blood cells/μL and 5 white blood cells/μL (Figure 2).

Three days post-surgery, the patient’s condition remained stable. Vital signs were normal, there was no fever, and inflammatory markers were within the normal range. The patient was then discharged and returned for a follow-up examination 2 weeks later. The patient reported no fever, back pain, abdominal pain, or other discomforts. Urinalysis at this time revealed light yellow urine, with 7 red blood cells/μL and 3 white blood cells/μL, both values falling within the normal range. Abdominal CT failed to detect any left ureteral stones, nor any complications such as perirenal hematoma, perirenal abscess, or ureteral stenosis (Figure 1D).

Upon a one-month follow-up call, the patient reported no discomfort and no recurrence of hematuria. The local hospital re-examined the urine routine and detected no red blood cells.

## Discussion

This case report illustrates a systematic diagnostic approach to gross hematuria, ultimately identifying an uncommon etiology: retrograde migration of a double-J stent from the left ureter through the renal vein into the inferior vena cava. The clinical significance lies in the successful endoscopic retrieval via ureteroscopy, which circumvented potential life-threatening complications including major vascular intervention or hemorrhagic sequelae. The post-procedural outcomes were remarkable. Hematuria resolved immediately, and the structured follow-up confirmed a sustained clinical improvement. Longitudinal monitoring demonstrated complete absence of procedure-related sequelae, with interval imaging verifying anatomical integrity and resolution of stent-induced vascular compromise.

Hematuria serves as a critical clinical sign that may signal an underlying pathological condition requiring urgent attention. Nevertheless, a thorough medical history, physical examination, and appropriately selected ancillary investigations often enable precise etiological diagnosis. A high index of suspicion combined with effective physician-patient communication facilitates evidence-based evaluation and management strategies (5, 6). In this case, the patient presented with hematuria. Through detailed historical analysis and consideration of the patient’s recent (1-day post-operative) double-J stent placement for urinary calculi, iatrogenic injury from the stent was prioritized in the differential diagnosis. Imaging studies confirmed retrograde migration of the double-J stent into the inferior vena cava, corroborating the clinical suspicion.

Initial evaluation of patients presenting with gross hematuria should prioritize three key domains: assessment of hemodynamic stability, identification of the underlying etiology, and establishment of unobstructed urinary drainage. Hemodynamic assessment constitutes the cornerstone of initial management, involving systematic evaluation of vital signs, physical examination findings, and hemoglobin/hematocrit levels. Hemodynamically unstable patients necessitate immediate resuscitative measures. Urological emergencies requiring urgent intervention include, but are not limited to, intraperitoneal bladder rupture, ureteroarterial fistula, and hemorrhagic cystitis. Conversely, painless gross hematuria in hemodynamically stable individuals typically warrants outpatient management. In such cases, timely referral to urology specialists for definitive evaluation is critical. Given the heterogeneous nature of hematuria etiologies, diagnostic strategies must be tailored to individual clinical contexts, with treatment modalities varying significantly based on underlying pathological processes.

Ureteral double-J stent placement is a cornerstone intervention in contemporary urological practice. Despite its widespread use, evidence-based timing for stent removal remains controversial. A large-scale case series identified 14–21 days as the optimal interval following renal transplantation (7). As utilization rates continue to rise, reported complication rates approximate 3.5% (8), with self-limiting manifestations including febrile episodes, hematuria, and urinary tract infections (9). However, serious life-threatening complications such as venous ectopia represent rare but critical exceptions. In 2002, Michalopoulos et al. documented a case of venous ectopia, highlighting this potentially fatal sequela (3). Literature review indicates relatively frequent occurrence of ectopic ureteral stent positioning, whereas perforation or laceration of urinary tract structures or adjacent organs constitutes an uncommon but life-threatening scenario. Maurice et al. characterized such injuries as Stenting-Induced Trauma (SIT), with an incidence rate of 0.11% (10). Venous ectopic migration of double-J stents falls under this SIT classification. Due to their low prevalence, venous (as opposed to cardiac) ectopias often present with atypical clinical manifestations, frequently misdiagnosed as routine post-procedural sequelae. Since 2012, heightened awareness and improved diagnostic modalities have led to an increasing number of reported cases, underscoring the need for vigilance in recognizing this complication.

When treating newly diagnosed gross hematuria patients, it is necessary to consider aspects like the patient’s surgical history, anticoagulation status, and catheter placement. Iatrogenic hematuria might result from intraoperative complications of uncertain origin, such as ureteral or bladder tears, thermal injuries, or over-inflation of the catheter balloon. Since certain medications can alter urine color and cause hematuria, a detailed review of the medication list is essential. Hematuria can sometimes cause a decline in hematocrit, necessitating blood transfusion, which may be seen in cases of trauma, ureteral arteritis, and hemorrhagic cystitis, thus these causes require urgent attention (11). Once the cause of hematuria is identified and the underlying problem is resolved, hematuria generally subsides in a short time. To safely perform ureteral stent insertion and prevent complications like double-J stent migration to the inferior vena cava, urologists can adopt the following measures: First, conduct a comprehensive preoperative evaluation, including detailed imaging studies like intravenous urography, CT urography, or ultrasound. Second, use fluoroscopy or ultrasound guidance during the stent insertion procedure, ensure the stent is sized appropriately for the patient’s ureter, and manipulate the stent gently during insertion to avoid trauma to the ureteral wall that could potentially lead to stent displacement (12). Third, closely monitor patients for any signs of complications after stent insertion.

## Limitations

This case report has several limitations. It is based on a single sample, which inherently lacks the statistical power and generalizability that multiple samples would provide. Additionally, the follow-up period was relatively short, potentially missing long-term outcomes and sequelae. Moreover, the condition described is extremely rare. Given these factors, further in-depth research, preferably involving larger sample sizes, extended follow-up durations, and more comprehensive data collection, is urgently required to improve our understanding of this condition.

## Conclusion

Hematuria is a common clinical presentation; however, venous ectopia induced by a double-J stent represents a rare yet significant complication. This case underscores the critical importance of considering venous ectopy in conjunction with potential bladder or ureteral injuries when evaluating post-stent hematuria. Prompt radiographic evaluation via computed tomography (CT) or ultrasound is essential to assess stent-related vascular risks prior to removal. Concurrently, close monitoring of hemodynamic parameters and hemoglobin/hematocrit levels is mandatory. In cases of hemodynamic instability or progressive anemia, urgent intervention, including endoscopic or surgical management, may be necessary to prevent life-threatening sequelae. This report highlights the need for heightened clinical suspicion and multidisciplinary collaboration in managing urological stent complications.

## Data Availability

The clinical data supporting the conclusions of this manuscript will be made available by the authors.
